# Less than one in four mothers get quality intrapartum health care services in Ethiopia

**DOI:** 10.1038/s41598-024-54506-x

**Published:** 2024-02-20

**Authors:** Wubshet Debebe Negash, Desale Bihonegn Asmamaw, Gizachew Tadesse Wassie, Abebaw Gedef Azene, Habitu Birhan Eshetu, Bewuketu Terefe, Kindie Fentahun Muchie, Getasew Mulat Bantie, Kassawmar Angaw Bogale, Tadele Biresaw Belachew

**Affiliations:** 1 Department of Health Systems and Policy, Institute of Public Health, College of Medicine and Health Sciences, University of Gondar, PO Box 196, Gondar, Ethiopia; 2 Department of Reproductive Health, Institute of Public Health, College of Medicine and Health Sciences, University of Gondar, PO Box 196, Gondar, Ethiopia; 3 Department of Epidemiology and Biostatistics, School of Public Health, College of Medicine and Health Sciences, Bahir Dar University, Bahir Dar, Ethiopia; 4 Department of Health Promotion and Health Behavior, Institute of Public Health, College of Medicine and Health Sciences, University of Gondar, PO Box 196, Gondar, Ethiopia; 5 Department of Community Health Nursing, School of Nursing, College of Medicine and Health Sciences, University of Gondar, PO Box 196, Gondar, Ethiopia; 6 Amhara National Regional State Public Health Institute, Bahir Dar, Ethiopia

**Keywords:** Quality, Intrapartum care, Ethiopia, Health care, Medical research

## Abstract

Intrapartum care is a platform of comprehensive healthcare for pregnant women that is designed to improve birth outcomes for mother and child. However, complications during the intrapartum period continued to be the leading cause of death for women of reproductive age and newborns. Therefore, the aim of this study was to assess the prevalence of quality of intrapartum care and its associated factors among mothers in Ethiopia. A community based cross sectional study was conducted among 4469 mothers who gave birth in the last 2 years. Quality of intrapartum care was analyzed based on the assessment of health facility delivery, skilled birth attendants and early initiations of breast-feeding. Stata version 14 software was used for data cleaning and analysis. A mixed effect multilevel logistic regression was conducted to determine factors associated with quality of intrapartum care. An adjusted odds ratio with 95% confidence interval and a *P* value of less than or equal to 0.05 was used for the identification of both individual and community level factors. Overall, the prevalence of quality intrapartum care in Ethiopia was 23.8% (95% CI 22.6, 25.13). Primary education (AOR = 1.46, 95% CI = 1.14, 1.88), rich household class (AOR = 1.48, 95% CI = 1.10, 1.98), history of ANC (AOR = 2.91, 95% CI = 2.18, 3.86), perceived distance to the health facility as not a big issue (AOR = 1.63, 95% CI = 1.30, 2.05), urban residence (AOR = 2.97, 95% CI = 1.93, 5.09), Tigray region (AOR = 5.01, 95% CI = 1.25, 20.59), community level poverty (AOR = 0.63, 95% CI = 0.41, 0.97), and having 2–4 children (AOR = 0.74, 95% CI = 0.56, 0.97) were significantly associated with quality of intrapartum care. The finding conclude that less than one in four mothers received good quality intrapartum care. In order to optimize the quality of intrapartum care, the government should empower women through extensive education. It is also recommended for the Ministry of Health to evaluate the health facilities and community health workers to increase coverage of ANC and provide financial assistance to rural residents and the poor household class.

According to the World Health Organization (WHO), intrapartum care is a platform of comprehensive healthcare for pregnant women that is designed to optimize birth outcomes for mother and child through the provision of skilled healthcare professionals in a well-maintained health care system^1^. There is a global transformation of maternal health from a survival objective to further improving the health of mothers and newborns^2,3^. Despite variations in the components of intrapartum care^1^, recent recommendations are placed on optimizing health and wellbeing for mothers and newborns during childbirth^4^. In conjunction with the provided clinical health care services, emotional support, good communication, and respectful care are recommended^5^.

The health of the woman and their child depends on the quality of care a woman received during the intrapartum period^6^. Pregnancy and childbirth related complications continue to be major sources of mortality among reproductive age women in lower and middle income countries^7^. Particularly, the intrapartum period is a time where the mother and the child become at high risk for death^8,9^. Globally, a total of 300,000 maternal deaths and five million perinatal deaths occur every year^10^. According to the World Health Organization report, 295,000 women died globally and 194,700 women died in sub-Saharan Africa as a result of pregnancy and childbirth in 2017. The maternal mortality ratio in Ethiopia was 267 in 2019, placing the nation 32nd out of 121 countries^11^.

Despite a decrease in child mortality, sub-Saharan Africa could not achieve Millennium Development Goal 4 because newborn deaths were largely disregarded and now account for 40% of under-five deaths^10,12^. Moreover, as compared to high-income settings, in the lower and middle income countries (LMICs), 23% of infant mortality and nearly half of stillbirths are due to intrapartum complications^13,14^. It is therefore critical to provide quality health care in order to get the greatest potential health outcome for the mother and newborns^15^. Since the major causes of maternal death and illness are preventable needing efforts of focusing on facility-based birth, skilled birth attendance, and rapid referral for emergency obstetric treatment if complications arise^16^.

In sub Saharan Africa and many low income countries, quality of intrapartum care remains a concern^17–19^. Related with quality of maternal and child health care services, a country representative studies were restricted on the assessment of quality antenatal care^20–22^. Although many primary studies were conducted in Ethiopia to determine the prevalence of quality of intrapartum care, so far, the studies were limited in a small geographical area and are inconclusive^23–28^. Moreover, these small studies were not considered the community level factors such as community level media exposure, education and poverty. Therefore, this study aims to fill the above research gaps by assessing the prevalence of quality of intrapartum care and its associated factors in Ethiopia.

## Methods

### Study setting, and design

The study was conducted in Ethiopia where it found in the horn of Africa. During the data collection period, the country was divided into nine regions named as: Tigray, Afar, Amhara, Oromia, Benishangul Gumuz, Gambela, Southern Nations Nationalities and People Region, Harari, and Somalia and two city administrations called Addis Ababa, and Dire Dawa. Zones are the next classifications, following the region. Then the Zone are divided in to Districts. The country has 118 million population, which is 14^th^ and 2^nd^ most populous country in the world and Africa, respectively^29^. Community based cross sectional study was conducted from January 18 to June 27/2016 among mothers who gave birth.

### Data collection procedures, tool and population

Even though Demographic Health Surveys (DHS) are conducted every 5 years, the recent available standardized data in Ethiopia is the 2016 Ethiopian Demographic Health Survey (EDHS)^30,31^. As a sampling frame, the 2007 Ethiopian population and housing census was used. The census was established into 645 enumeration areas: 202 urban and 443 rural. Then, a fixed number of 28 households per cluster was selected by equal probability systematic sampling technique^32,33^. All mothers who are members of the household and visitors who slept in the household the night before the survey were eligible for the interview^31^. The detailed of the methods for the survey is available elsewhere^34^.

Those mothers who were available during the survey in Ethiopia were the source population. Whereas, those mothers who were in the selected enumeration area were considered as study population and used for analysis.

### Study variables

The dependent variable of the study was Quality of intrapartum care which was determined by the combination of the following three interventions: giving birth at the health facility, birth assisted by skilled personnel, and new born begin to breastfeed within 1 h of birth^4,35^. Skilled birth attendant (doctors, health officers, nurses, and midwifes) refers to provider of delivery during labour. When a woman received care from numerous providers, the most skilled provider was recorded^22^. Those mothers who reported the aforementioned skilled birth attendant, birth at health facility, and newborn begin breast feeding within 1 h were considered as getting quality intrapartum care and coded as 1 otherwise no quality of intrapartum care and was codded as 0.

All the independent variables were included after a thorough review of literatures^23–28^. Both individual and community level factors were considered for this study. Age: 15–24, 25–34 and 35–49 years; educational status: no formal education, primary education, secondary and higher education; current marital status: married, unmarried: individual level media exposure; yes (if the mother watches television, or listen radio, or reads a newspaper/magazine at least once a week), otherwise no; history of ANC: yes, no; age at first birth: before 18, 18 or above were considered as individual level variables. Whereas, residence (urban vs rural); distance to the health facility (big problem vs not big problem), community level poverty, community level education and community level media exposure from the community level factors were considered for this particular study. Community level poverty was generated by the proportion of poorest and poorer wealth classes. Community level education was calculated by the proportion of primary, secondary and higher education. Similarly, community level media exposure was generated by the proportion of media exposure. The data for all the three community level variables were not normally distributed. Therefore, each variables were categorized as “low” if the proportion was below 50%, “high” for including median and above the median value^22,36^.

### Data management and analysis

Stata version 14 software was used for data extraction, cleaning and analysis. Initially, the sample data to be representative, weighting was conducted. We used the individual weight for women (v005) and the individual sample weight was generated by dividing (v005) by 1,000,000 before analysis to approximate the number of cases^33^. Secondly, proportion of quality intrapartum care across each independent variable was analyzed. All the variables having a *P* value less than 0.05 were used for the multivariable analysis. Adjusted odds ratio with 95% confidence interval and a p value of 0.05 were used for identifications of associated factors. Finally, multilevel analysis having both the random (presented with ICC) and fixed effect (expressed by adjusted odds ratio) were done.

Demographic health survey data is hierarchical in nature where individual mothers are nested within the communities. As a result the assumption of independency might be violated in logistic regression. Hence, it could be better to assess both the individual and community level variables on the quality of intrapartum care^37^. Multilevel analysis having four models were analyzed. Deviance (− 2loglikelihoods) was used for model comparison.

### Ethical approval

The ethical approval and permission to access the data were obtained from the DHS website www.measuredhs.com. All methods were approved by ICF International and an Institutional Review Board (IRB) in Ethiopia, in accordance with United states Department of Health and Human Services requirements for human subject protection. Ethical clearance was obtained by the Institutional Review Board of Demographic and Health Surveys (DHS) program data archivists after the consent manuscript was submitted to DHS Program/ICF International. Informed consent was obtained from all subjects and/or their legal guardian(s) of minors age below 16. No information obtained from the data set was disclosed to any third person. The study is not experimental study. Further explanation of how the DHS uses data and its ethical standards can be found at: http://goo.gl/ny8T6X.

## Results

### Distribution of quality intrapartum care across individual and community level factors

Higher (26.32%) prevalence of quality intrapartum care was observed among mothers in the age group of 15–24 years. More than half (58.71%) of secondary educated mothers received quality intrapartum care. Of those mothers who had ANC history, 32.57% had reported quality intrapartum care (Table 1).

**Table 1:** Distribution of quality intrapartum care across individual level factors in Ethiopia, EDHS 2016 (n = 4469).

Variable	Categories	Frequencies	Percentage	Quality of intrapartum care	Chi2
Age	15–24	1295	28.99	26.32	0.00
	25–34	2288	51.2	23.76	
	35–49	886	19.82	20.46	
Education of mother	No formal	2704	60.51	15.42	
	Primary	1611	36.06	34.70	
	Secondary and higher	154	3.44	58.71	
Occupation	Working	1874	41.92	25.29	0.00
	Not working	2595	58.08	22.83	
Marital status	Married	4258	95.29	23.80	0.005
	Not married	211	4.71	25.08	
Religion	Orthodox	1532	34.29	30.32	0.00
	Catholic	43	0.97	7.90	
	Protestant	904	20.23	22.03	
	Muslim	1874	41.92	20.85	
	Other*	116	1.02	18.48	
Wealth index	Poor	2022	45.25	13.94	0.00
	Middle	930	20.81	20.50	
	Rich	1517	33.94	39.14	
Media exposure	Yes	1549	34.66	34.85	0.00
	No	2920	65.34	18.03	
Husband education	No formal	1945	45.68	15.32	0.00
	Primary education	2031	47.69	27.48	
	Secondary and higher	283	6.64	55.76	
Age at first birth	Less than 18	1654	37.02	19.48	0.00
	18 and above	2815	62.98	26.44	
Number of living children	1	972	21.76	35.73	0.00
	2–4	2061	46.11	24.83	
	5 and more	1436	32.12	14.44	
History of ANC	Yes	1519	66.02	32.51	0.00
	No	2950	33.98	7.06	

*Traditional, unknown.

### Distribution of quality intrapartum care across the community level factors

Of urban resident mothers, 61.41% reported quality intrapartum care. Among high media exposed mothers, 32.38% were reported the quality of their intrapartum care (Table 2).

**Table 2:** Distribution of quality intrapartum care across the community level factors in Ethiopia, EDHS 2016 (n = 4469).

Variables	Categories	Frequencies	Percentage	Quality of intrapartum	Chi2
Place of residence	Urban	542	12.12	61.41	0.00
	Rural	3927	87.88	18.68	
Distance to the health facility	Not big problem	1765	39.49	35.67	0.00
	Big problem	2704	60.51	16.5	
Community level media exposure	High	2327	52.08	32.38	0.00
	Low	2142	47.92	14.61	
Community level poverty	High	1721	38.50	13.99	0.00
	Low	2748	61.50	30.44	

#### Individual and community level factors associated with quality of intrapartum care

In model I (adjusted for individual level factors), age 35–49 years (AOR = 1.69, 95% CI = 1.15, 2.52), mothers primary education (AOR = 1.68, 95% CI = 1.32, 1.13), rich wealth class (AOR = 2.21, 95% CI = 1.68, 2.89), 2–4 number of children (AOR = 0.76, 95% CI = 0.58, 0.99), and history of ANC (AOR = 3.38, 95% CI = 2.54, 4.48) were significantly associated with quality of intrapartum care. Whereas, urban residence (AOR = 4.02, 95% CI = 2.38, 6.81), perceived distance as not a big problem (AOR = 1.68, 95% CI = 1.35, 2.10), community level media exposure (AOR = 1.61, 95% CI = 1.07, 2.40), community level poverty (AOR = 0.48, 95% CI = 0.31, 0.73), community level education (AOR = 2.48, 95% CI = 1.67, 3.17) and region were significantly associated with quality of intrapartum care.

In the final model, having primary education, rich household wealth index, having two or more number of living children, and history of ANC from the individual level factors and urban residence, distance as not big problem and community level education from the community level factors were significantly associated with quality of intrapartum care.

Accordingly, the likelihood of quality intrapartum care was 1.46 [AOR = 1.46, 95% CI = 1.14, 1.88] times higher among mothers who had attended primary education as compared to those mothers who had not followed formal education.

Those mothers from the rich wealth index class had 1.48 times higher likelihood of getting quality intrapartum care as compared to mothers from the poor household wealth status (AOR = 1.48, 95% CI = 1.10, 1.98). Similarly, the odds of quality of intrapartum care was 0.37 times lower among those mothers who were from population of high proportion of community level poverty (AOR = 0.63, 95% CI = 0.41, 0.97).

The odds of quality of intrapartum care was 0.26 (AOR = 0.74 95% CI = 0.56, 0.97) and 0.46 (AOR = 0.54, 95% CI, 0.36, 0.81) times lower among those mothers who had 2- 4 and 5 or more children as compared with those mothers who had no children at all, respectively.

The likelihood of getting quality intrapartum care was 2.91 (AOR = 2.91, 95% CI = 2.18, 3.86) times higher among mothers who had history of ANC as compared to those mothers who had no history of ANC.

Those mothers who perceived distance from the health facility as not a big problem had 1.63 times higher likelihood of getting quality intrapartum care than those mothers who perceived distance from the health facility as a big problem (AOR = 1.63, 95% CI = 1.30, 2.05).

Urban resident mothers had 2.97 times higher likelihood of getting quality intrapartum care (AOR = 2.97, 95% CI = 1.93, 5.09) as compared to their counterparts of rural resident mothers.

Compared with Afar region, the odds of quality of intrapartum care was 5 times higher among mothers who were from Tigray region (AOR = 5.01, 95% CI = 1.25, 20.59) (Table 3).

**Table 3:** Multilevel analysis to identify individual and community level factors of quality of intrapartum care in Ethiopia, EDHS 2016 (n = 4469).

Variables	Categories	Intrapartum quality care		Model 1 AOR (95% CI)	Model 2 AOR (95%CI)	Model 3 AOR (95%CI)
		Yes n (%)	No n (%)			
Age in years	15–24	342 (26.37)	954 (73.63)	1		**1**
	25–34	544 (23.76)	1744 (76.24)	1.21 (0.91, 1.59)		1.42 (0.85, 1.47)
	35–49	181 (20.46)	704 (79.54)	1.69 (1.15, 2.52)		1.17 (0.99, 2.20)
Educational status of mothers	No formal	417 (15.42)	2287 (84.58)	1		1
	Primary	559 (34.70)	1052 (65.3)	1.68 (1.32, 2.13)		**1.46 (1.14, 1.88)**
	Secondary and higher	90 (58.71)	63 (41.29)	1.52 (0.87, 2.67)		1.09 (0.61, 1.92)
Occupation of the mothers	Working	474 (25.29)	2003 (77.17)	1.04 (0.85, 1.28)		1.05 (0.85, 1.29)
	Not working	593 (22.83)	1400 (74.71)	1		1
Husband education	No formal education	298 (15.37)	1647 (84.68)	1		1
	Primary	558 (27.48)	1473 (72.52)	1.28 (1.03, 1.62)		1.22 (0.97, 1.55)
	Secondary and higher	158 (55.76)	125 (44.24)	1.95 (1.26, 3.05)		1.54 (0.98, 2.42)
Media exposure	No	526 (18.03)	2393 (81.97)	1		1
	Yes	540 (34.85)	1009 (65.15)	1.04 (0.82, 1.31)		0.85 (0.67, 1.07)
Wealth index	Poor	282 (13.94)	1740 (86.06)	1		1
	Middle	191 (20.50)	739 (79.50)	1.11 (0.84, 1.46)		1.02 (0.76, 1.35)
	Rich	594 (39.14)	923 (60.86)	2.21 (1.68, 2.89)		**1.48 (1.10, 1.98)**
Age at first birth in years	< 18	322 (19.48)	1332 (80.52)	1		1
	≥ 18	744 (26.44)	2070 (73.56)	0.86 (0.69, 1.08)		0.85 (0.68, 1.06)
Number of living children	1	347 (35.73)	625 (64.27)	1		1
	2–4	512 (24.83)	1549 (75.17)	0.76 (0.58, 0.99)		**0.74 (0.56,0.97)**
	≥ 5	207 (14.44)	1228 (85.56)	0.49 (0.33, 0.73)		**0.54 (0.36,0.81)**
History of ANC	Yes	959 (32.51)	1991 (67.49)	3.38 (2.54, 4.48)		**2.91 (2.18, 3.86)**
	No	107 (7.06)	1411 (92.94)	1		1
Place of residence	Urban	333 (61.41)	209 (38.59)		4.02 (2.38, 6.81)	**2.97 (1.93, 5.09)**
	Rural	734 (18.68)	3193 (81.32)		1	1
Distance to the health facility	Not Big problem	630 (35.67)	1135 (64.33)		1.68 (1.35, 2.10)	**1.63 (1.30, 2.05)**
	big problem	437 (16.15)	2267 (83.85)		1	1
Community level media exposure	High	754 (32.38)	1574 (67.62)		1.61 (1.07, 2.40)	1.49 (0.99, 2.25)
	Low	313 (14.61)	18,289 (85.39)		1	1
Community level poverty	High	241 (13.99)	1480 (86.01)		0.48 (0.31, 0.73)	**0.63 (0.41, 0.97)**
	Low	826 (30.04)	1923 (69.96)		1	1
Community level education	High	825 (35.15)	1523 (64.85)		2.48 (1.67, 3.71)	**1.71 (1.14, 2.55)**
	Low	241 (11.36)	1879 (88.64)		1	1
Region	Tigray	175 (54.43)	147 (45.57)		6.62 (1.69, 25.8)	**5.01 (1.25, 20.59)**
	Amhara	619 (75.67)	198 (24.33)		2.56 (0.66, 9.89)	2.04 (0.51, 8.21)
	Oromia	1636 (82.31)	352 (17.69)		1.71 (0.45, 6.56)	1.85 (0.46, 7.36)
	Somali	159 (82.95)	33 (17.05)		2.34 (0.57, 9.65)	2.72 (0.63. 11.67)
	Benshangul Gumuz	36 (77.06)	11 (22.94)		3.14 (0.64, 15.35)	2.64 (0.52, 13.48)
	SNNPR	679 (74.75)	229 (25.25)		3.22 (0.84, 12.39)	2.75 (0.68, 11.02)
	Gambela	7 (64.76)	4 (35.24)		2.78 (0.35, 22.35)	2.16 (0.25, 18.88)
	Harari	6 (51.67)	5 (48.33)		5.28 (0.68, 41.31)	4.88 (0.58, 40.47)
	Addis Ababa	41 (36.30)	72 (63.70)		2.44 (0.58, 10.11)	1.87 (0.43, 8.11)
	Dire Dawa	9 (45.93)	10 (54.73)		10.42 (1.67, 64.69)	**7.71 (1.17, 50.74)**
	Afar	38 (87.73)	5 (12.27)		1	1

Significant values are in [bold].

Statistically significant at *P* value < 0.05.

AOR, Adjusted odds ratio; COR, Crude odds ratio; Null model, Without factors; Model 1, Adjusted for individual-level factors; Model 2, Adjusted for community-level factors; Model 3, Adjusted for both individual and community-level factors.

### Random effects (measures of variation) results

The Intra class correlation coefficient for the null model was 0.4796, which means that 47.96% of quality of intrapartum care variation among mothers was due to differences in clusters. Final model which had lowest deviance was selected as the best fitted model. The heterogeneity of quality of intrapartum care was expressed by median odds ratio. The final model indicated that the odds of quality of intrapartum care was 3.01 times higher among mothers who were from higher risk of quality intrapartum care as compared with those mothers who were from lower risk cluster. Regarding the proportional change in variance (PCV), 53.46% of quality intrapartum care was explained by model four (Table 4).

**Table 4:** A measure of variation for quality of intrapartum care in Ethiopia, EDHS 2016 (n = 4469).

Measures of variation	Null model	Model 1	Model 2	Model 3
Variance	3.03	1.67	1.54	1.41
MOR	4.49	3.34	3.21	3.01
PCV (%)	Ref	44.88	49.17	53.46
ICC (%)	47.96	33.65	32.02	29.98
Model fitness				
Deviance (− 2*Likelihood)	3985.5852	3515.6278	3711.0556	3394.268

*MOR* Median odds ratio, *PCV* Proportional change in variance, *ICC* Intra class correlation coefficient.

## Discussion

Findings of this research revealed that 23.8% (95% CI 22.6–25.13) mothers had experienced good quality of intrapartum care. The prevalence implies that more than three-fourths of the mothers did not received quality intrapartum care. The finding is lower than studies conducted at North Achefer, Ethiopia (27.3%)^38^, Tigray (29.2%)^39^, Jimma zone, Ethiopia (74.9%)^40^. The difference may accounted that the study setting where the current study was conducted based on country representative data. Whereas, the previous studies were conducted in limited geographical area at health facilities. The other possible reason for the difference may be due to quality assessment methods; the current study used three key variables which are found in the EDHS, whereas the previous studies used checklists which hold additional variables such as stages of labour, and availability of health services.

The finding is higher than study conducted in Jabi tehnan district (13%)^41^. The lower prevalence of good quality of intrapartum care might be differences in the assessment method where the study conducted in Jabi Tehinan district in North West Ethiopia, was evaluated the quality of intrapartum care by asking the women about their experience of resources, their cognition, respect, dignity and equity of care they received.

The likelihood of quality intrapartum care was higher among mothers who had learned primary education as compared to those mothers who had no formal education. The finding is similar studies conducted in Uganda^42^, Cameroon^43^, Nigeria^44^ and Kenya^45^. More educated mothers had higher likelihoods of understanding the benefits of quality health care, being aware of pregnancy-related morbidities and mortality, perhaps because they are more likely to seek medical care^46,47^. In view of this finding, education is an indicator that needs to be improved to better enable women to access maternal health care.

Those mothers who were from the rich wealth class had higher likelihood of getting quality intrapartum care as compared to the poor wealth class^48,49^. Similarly, the odds of quality of intrapartum care was lower among mothers who were from population of high community level poverty. The finding is similar with studies conducted in Nepal^50^, Kenya^51^, and East Africa^52^. This is because low wealth class women can have specific worries and feels inadequate for service, which in turn lowers the minimum level of health care service^53,54^. Furthermore, the cost of travelling to distant health facilities contributes indirectly to the cost of quality intrapartum care, which can be easily covered by pregnant women from rich wealth class households^55^. Therefore, economic variations in obtaining maternal or reproductive health services plays a role in receiving quality health care services^56,57^.

The odds of getting quality intrapartum care is higher among mothers who were from the urban resident as compared with their counterparts of rural resident mothers. The finding is similar with elsewhere study in Bangladesh^46^. The reason for low quality of intrapartum care in rural area might be accounted due to barriers such as transportation, and availability of health facilities^47^. Conditionally mothers from rural area may face difficulties such as long distance to reach the health facilities and poor road conditions as compared with mothers from urban residence^58^.

The odds of quality of intrapartum care was lower among those mothers who had two or more children as compared with those mothers who had no children at all. In line with this, findings from Cameroon^43^ and Sudan^59^ revealed that those mothers who had more children were less likely to utilize health care services. Those mothers who had more children may sense overconfidence from their experience of past pregnancy^60^.

The likelihood of getting quality intrapartum care was higher among mothers who had history of ANC as compared with those mothers who had no history of ANC. The same finding was observed in Ethiopia^61^, and Rwanda^62^. The possible reason might be that those mothers who had history of ANC had higher likelihood of getting extensive health education sessions, skilled birth attendant and know quality of intrapartum care and report it positively^63,64^.

Those mothers who perceived distance from the health facility as not a big problem had higher likelihood of getting quality intrapartum care than those mothers who were perceived distance from the health facility as a big problem. In congruent with this studies in Uganda^65^ and Tanzania^66^ revealed the same result. These findings indicated that improving access to health care services as well as distribution of health services, particularly in distant locations, should be prioritized.

The odds of quality of intrapartum care was higher among mothers who were from Tigray region compared with Afar region. The lower odds of quality intrapartum care in Afar region might be due to the reason that the region is highly affected by drought and disaster. Moreover the Afar region is lower socioeconomic status as compared to Tigray region^67^. This implies that strong attention is needed for disadvantaged areas of Ethiopia.

The study implied that out of the four mothers, only one was delivered at the health facilities, assisted by skilled birth attendants and initiated breast feeding within 1 h. This means that hundreds of mothers had not received all components of intrapartum care. Hence, to counteract the problem, the Ministry of Health and health facilities need to continuously evaluate and improve maternal health care services.

### Strengths and limitations

Adequate sample size and nationally representative data was used. The study was considered both the individual and community level factors and accounted the hierarchical nature of the data. The study have the following limitations; first the cross sectional nature of the data causes to impossible to identify cause effect relationship. Some key factors such as labour, were not included in the analysis of quality intrapartum care where the level of quality intrapartum care may be low.

## Conclusion

The finding revealed that less than one in four mothers had get quality intrapartum care which is minimal as compared to other studies. Individual level factors of mothers’ primary education, rich household class, had more than two number of children were statistically significant factors of quality intrapartum care. Whereas, urban residence, distance as not a big problem and community level education from the community level factors were significantly associated with quality of intrapartum care. Therefore, to improve quality of intrapartum care, empowerment of mothers through extensive women education, and strengthening of ANC coverage, particularly for rural resident women are important recommendations.

## Data Availability

The data used in this study were publicly available. The data set can be found in the following website: https://dhsprogram.com/data/dataset/Ethiopia_Standard-DHS_2016.cfm.

## Abbreviations

AOR: Adjusted odds ratio
DHS: Demographic Health Survey
EAs: Enumeration areas
EDHS: Ethiopian Demographic and Health Survey
ICC: Intra-class correlation coefficient
MOR: Median odds ratio
PCV: Proportional change in variance
SNNPR: South Nations, Nationalities, and Peoples’ Region
WHO: World Health Organization
